# Exclusion or exemption from risk regulation? A comparative analysis of proposals to amend the EU GMO legislation

**DOI:** 10.15252/embr.202051061

**Published:** 2020-11-29

**Authors:** Tomasz Zimny, Dennis Eriksson

**Affiliations:** ^1^ Institute of Law Studies Polish Academy of Sciences Warsaw Poland; ^2^ Department of Plant Breeding Swedish University of Agricultural Sciences Alnarp Sweden

**Keywords:** Synthetic Biology & Biotechnology, S&S: Economics & Business, Plant Biology

## Abstract

The EU Court of Justice’s decision that gene‐edited plants should be regulated as GMOs triggered various proposal to amend the EU Directive on the release of GMOs in the environment.

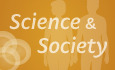

In the EU, the legal status of agricultural products resulting from the use of new breeding techniques (NBTs)—among others the new gene‐editing technologies—has been subject to dispute even before the Court of Justice of the EU (CJEU) ruled that products of newer forms of mutagenesis should be regulated as genetically modified organisms (GMOs; Breyer *et al*, 2009; Abbott, 2015). In November 2019, the Council of the EU requested the European Commission (EC) to submit a study, and a proposal if appropriate, for addressing the legal status of novel genomic techniques under Union law, and this will likely provide more clarity for the products of NBTs. In the meantime, several proposals for amending the current GMO legislation have been published. We here provide an analysis of their respective key features, similarities and differences, and potential implications of their adoption.

## Introduction

Much of the dispute on the legal status of NBT products has centred on the scope of the GMO definition. According to article 2(2) of the Directive 2001/18/EC (hereafter: the directive), a GMO is defined as “an organism, with the exception of human beings, in which the genetic material has been altered in a way that does not occur naturally by mating and/or natural recombination” (Directive, 2001). This definition is further supplemented in Annex I A Part 1 by a non‐exhaustive list of techniques that result in a GMO. Additionally, Annex I A Part 2 comprises an exhaustive list of techniques that are not considered to result in a GMO, whereas Annex I B includes techniques, the application of which results in genetic modification, but whose products are exempted from the provisions of the directive. The latter include mutagenesis and cell fusion of organisms that can exchange genetic material through traditional breeding methods. The exemptions were apparently made because products of these techniques had already been on the market for many years and with a long safety record (Directive, 2001).

The exemptions were apparently made because products of these techniques had already been on the market for many years and with a long safety record.

The scope of these exemptions has been subject to plenty of discussions, in particular regarding the products of genome editing since many of these are the result of what is scientifically considered as mutagenesis. The concern is whether the exemption applies only to techniques conventionally used when the directive was adopted in 2001 or if it also covers more recently developed techniques, such as genome editing through site‐directed mutagenesis. The CJEU decided in Case C‐528/16 (CJEU 2018) that only organisms obtained by techniques that have conventionally been used and have a long safety record are exempted from the scope of the directive. The position of the court is widely interpreted as resulting in treating all gene‐edited organisms in the EU as regulated GMOs, regardless of the nature of the change in their genomes—whether they feature point mutations or introduction of large fragments of DNA, though there are also alternative and more nuanced interpretations.

The position of the court is widely interpreted as resulting in treating all gene‐edited organisms in the EU as regulated GMOs, regardless of the nature of the change in their genomes…

The judgement inspired several stakeholders to publish proposals for amending the current GMO legislation, though some were published before 2018. Other earlier proposals have suggested a complete elimination of the current legal framework for GMOs in the EU and an entirely novel approach focused on a trait‐oriented framework. These proposals are unrealistic in the current political context—that is, it will be impossible to build a political majority for such an approach—and therefore not included in our analysis. Some proposals are rudimentary and simply argue that the legislation should be changed as it is outdated or has unacceptable consequences, while others constitute standalone, detailed legislative proposals. Using the presence of concrete and elaborate details for legal amendment as a criterion, we have selected six proposals (Table 1) for analysis.

**Table 1:** The analysed legislative proposals and their respective main characteristics.

No.	Proponent	Nature of deregulation	Scope of deregulation	Formal approval	Type of institution
1	Deutsche Akademie der Naturforscher Leopoldina (Leopoldina *et al,* 2019)	Change in definition: only organisms containing recombinant DNA are GMOs. Null segregants, products of SDN1 and SDN2 and cisgenesis are not GMO	All organisms	Confirmation of status	Academic institution
2	Association Française des Biotechnologies Végétales/Wissenschaftlerkreis Grüne Gentechnik e.V. (AFBV & WGG, 2020)	Exclusion of null segregants. Exemption of products of SDN1 and SDN2, and cisgenesis/intragenesis.	All organisms (but the authors claim that they had plants in mind)	Confirmation of status	NGO
3	The Government of the Kingdom of Netherlands (The Government of Netherlands, 2017)	Exemption of plants being products of SDN1 and SDN2, cisgenesis/intragenesis and null segregants.	Plants	Justification after the release, at the behest of the European Commission or member state	Government
4	Deutsche Akademie der Naturforscher Leopoldina (Leopoldina *et al,* 2019)	Exemption of null segregants, products of SDN1 and SDN2 and cisgenesis.	All organisms	Confirmation of status	Academic institution
5	Grow Scientific Progress (European Citizen’s Initiative, 2019)	Exemption of various products based on a pre‐approved list of traits known to exist in the past.	Organisms featuring predefined traits	Notification procedure	Group of students
6	A coalition of Norwegian experts coordinated by the Norwegian Biotechnology Advisory Board (Bratlie *et al*, 2019)	Tiered approach: Exclusion of null segregants; products of genome editing that could be achieved during conventional breeding subject to notification; products of cisgenesis/intragenesis subject to expedited approval.	All organisms	Depending on nature of the changes	Expert body

GMO, genetically modified organism; NGO, non‐governmental organisation; SDN, site‐directed nuclease.

## Overview of the proposals

All of the analysed proposals envisage an amendment rather than a total overhaul of the GMO legislation, which is understandable given the apparent urgency of the situation, in particular the fact that the current EU GMO legislation does not seem to be harmonised with that of the major trade partners. Nevertheless, even a minor change in the legislation may have significant consequences. We have divided the analysed proposals into two major groups: proposals to alter the definition of a GMO and proposals that amend the list of GMOs that are exempted from the scope of the legislation.

All of the analysed proposals envisage an amendment rather than a total overhaul of the GMO legislation, which is understandable given the apparent urgency of the situation…

The German Leopoldina Academy (proposal 1) (Leopoldina *et al,* 2019) suggests either a direct change of the definition (art. 2(2)) or an indirect change via the annex I A part 2 (organisms not considered to be a GMO). The term “GMO” should refer to an organism, whose genetic material “(…) is altered in the shape of insertion of genetic information into the genome in a way that does not occur naturally”. The consequences would be twofold. First, the change of the expression “has been altered” to “is altered in the shape of insertion of genetic information” is meant to remove an ambiguity in the current definition, as it remains a point of debate whether the currently used term refers to the resulting genetic feature of the organism or to the process by which the organism’s genetic material has been modified. Second, the addition of “insertion of genetic information” limits the scope of the term “GMO” by excluding organisms featuring deletions, regardless of size.

The proposal by AFBV and WGG (proposal 2) (AFBV&WGG, 2020) also includes a change of the definition of GMO, through art. 2(2) and the Annex I A part 1, by classifying genome editing as a technique leading to genetic modification and by excluding null segregants from the scope of the directive. The proposal also includes amendments to the exemptions from the scope of the directive (new Annex I C).

The remaining examined proposals do not redefine the legal term “GMO”, but aim at limiting the scope of the directive by changing contents of Annex I B or introducing new annexes. Such a change would generally result in treating products of the techniques listed there as GMOs that are exempted from the provisions of the directive.

According to the proposal of the Dutch Government (proposal 3) (The Government of Netherlands, 2017), exempted GMO products are those resulting from conventional mutagenesis and cell fusion, similar to the current legislation. In addition, plants resulting from techniques which do not involve the introduction of other genetic material than material from the same or crossable plant species, as well as plants in which recombinant DNA that was used for modification are no longer present, should also be exempted.

Another proposal by the Leopoldina (proposal 4, provided in case the aforementioned changes in the definition, i.e. proposal 1, would not be accepted) exempts “targeted molecular techniques which, when applied, effect a genetic modification that may have occurred naturally”. Examples are techniques that cause deletions of DNA; exchange individual base pairs; do not cause stable insertion of genetic information; or cause the insertion, inversion or translocation in the genome of genetic information known to occur; or can occur with high probability in the natural gene pool of the same species or closely related species.

In addition to amendments of the GMO definition, proposal 2 also features addition of a point 4 to the current Annex I A part 1 (techniques leading to genetic modification), where genome editing techniques are described as “a group of technologies that allow the targeted modification of genetic information by adding (insertion of), removing (deletion of), or exchanging (replacement of) nucleotides at a specific location in the genome of the recipient organism”. The proposal also features a two‐part addition to Annex I C. The first part lists plants that would be excluded from the scope of the new directive, generally plants with alleles edited so that they reproduce functionalities present in their natural gene pools or functionalities that can be obtained by spontaneous or induced mutagenesis or plants with genes from their natural gene pool inserted at a particular site. The second part of the new Annex I C describes a new confirmation procedure, through which the applicant would obtain official confirmation that their product is not regulated.

A proposal from “Grow Scientific Progress” (proposal 5; European Citizen’s Initiative, 2019) introduces a clear distinction between conventional mutagenesis, the products of which are exempted according to Annex I B, and other types of genetic modification, some of which would be exempted according to a newly created Annex I C. These exemptions would apply to techniques that result in modifications that could have been obtained by traditional breeding methods or methods, “including via breeding with other species with which the resulting organism could naturally exchange genetic material. Apart from this, proposal 5 also includes details that are not present in any of the other proposals: the idea of a rigid definition of a long safety record; and the concept of “traditional breeding methods”. It also introduces a mechanism of subjecting products of conventional mutagenesis to risk assessment, should a novel trait render the resulting organism a risk to human health or the environment—which is currently missing from the GMO legislation, since all products of conventional mutagenesis are considered safe and not subject to specific risk assessment.

Bratlie *et al* (2019; proposal 6) introduce a tiered system, whereby organisms with temporary, non‐heritable changes would be exempted from the scope of the legislation, while “genetically engineered organisms with changes that exist or can arise naturally and can be achieved using conventional breeding methods” would be subject only to a notification procedure and confirmation that they meet the aforementioned criteria. This tier “would apply to GMOs with genetic changes that can also be obtained by conventional methods, including substitution of an allele with another one that already exists within the species, or mutations that can arise naturally or by mutagenesis”. Products of cisgenesis would generally fall into tier 2, subjected to expedited approval, while organisms with genetic changes that cross species barriers or that involve artificial DNA would be subject to a full authorisation procedure including risk assessment. The authors also propose that all GMOs should be subject to assessment of “societal benefit, sustainability and ethics”, as is the case with the current GMO authorisation procedure in Norway.

The presented proposals have various features in common. All generally strive to address the lack of clarity associated with the regulatory status of the products of NBTs. The similarity between the Dutch proposal from before the CJEU’s judgement and the others is a good example of how the Court’s decision stimulated activity in this respect, even though the regulatory status of such organisms was not clear before the court decision. The analysed proposals also aim at a step towards liberalisation in the legislation by either excluding or exempting generally described group(s) of organisms from the provisions of the directive.

## Exclusion versus exemption

The most significant difference between the analysed proposals is the distinction between amending the GMO definition and expanding the list of exemptions. This would have profound consequences: an organism which is not classified as a GMO, has the same legal status as an organism developed through any conventional technique. This means that member states have very limited, if any, means for restricting its development or commercialisation. By contrast, an exemption does not protect the products of an exempted technique from being subjected to national restrictions, as ruled by the CJEU. In fact, this right has already been exercised: in a recent judgement, the French Conseil d’État mandated that plants developed through random mutagenesis performed on *in vitro* cultures shall be subject to the legal provisions that apply to GMOs, rather than being exempted as all products of random mutagenesis are at the EU level (https://www.conseil‐etat.fr/ressources/decisions‐contentieuses/dernieres‐decisions‐importantes/conseil‐d‐etat‐7‐fevrier‐2020‐organismes‐obtenus‐par‐mutagenese). This judgement is currently in the process of being implemented into French national law. As such, changes in the directive may either provide for legal certainty and stability for breeders or may become a source of another set of disputes as to whether a particular product will be regulated. The difference between an exclusion and an exemption may therefore have serious consequences for academia and industry.

… an exemption does not protect the products of an exempted technique from being subjected to national restrictions, as ruled by the CJEU.

Similarly, an amendment may influence the possibility of conducting field trials. If a plant is considered to be a regulated GMO, any field trials are regulated by national provisions on the basis of the Directive. These procedures are costly—significantly so for public researchers with minor budgets—and, depending on the country, rather restrictive. Excluding groups of organisms from the directive via a redefinition of GMO would therefore impact field trials as well, whereas exempting those organisms would again allow member states to introduce national restrictions.

Deregulation of certain groups of organisms would also influence the innovation potential of academia. Researchers would know that certain organisms or the use of certain techniques would be more easily transferable to the commercial sector than regulated ones. Again, given the potential of national restrictions, the difference between exclusions and exemptions is clearly visible, since any of the exempted organisms may still face national restrictions.

## Scope of proposed deregulation

Another aspect is the biological scope of planned exclusions or exemptions. The discussion in general seems to be revolving about potential deregulation of plants, in particular crop species used in agriculture. Hence, some of the proposals—in particular proposals 2, 3, 5—limit the scope of the deregulation, either by indicating particular groups of organisms or even particular traits. The remaining proposals do not contain such restrictions. The biological scope of deregulation could be a “deal breaker” when it comes to assessing the conformity of proposed solutions with the precautionary principle. GM plants may spread, for instance through pollen flow, but highly selected crop species usually require high maintenance and would likely not be competitive in the natural environment. Hence, one might argue that the deregulation of such crop plants would not necessarily elevate environmental risks above acceptable levels. If, in contrast, the deregulation would apply to all organisms, it could be argued that in many cases—gene‐edited insects, fish and other organisms—their spread in the environment could lead to potential risks that would be difficult to control. One has to bear in mind that laws have general application: once enacted, there is little room for restrictive or narrow interpretation. As such, the scope of deregulation, that is which organisms, regardless of technique, will be deregulated, should be taken into consideration.

The biological scope of deregulation could be a “deal breaker” when it comes to assessing the conformity of proposed solutions with the precautionary principle.

If a group of organisms were to be excluded from the scope of the legislation, then such organisms would be treated in the same way as conventionally bread organisms. In particular, such organisms would not be subjected to risk assessment and authorisation procedures, which would improve the commercial potential of techniques through which such excluded or exempted organisms are produced and broaden the spectrum of technologies EU breeders can use. Currently, developing organisms that face GMO authorisation procedures constitutes an investment risk, given the uncertain outcomes of these procedures, their cost and length, and possible national restrictions by member states.

Furthermore, food and feed products developed from such organisms would not have to be labelled as GMOs. This would have consequences for the farmers too. Since the accidental admixture thresholds (currently up to 0,9%) and unauthorised GMO thresholds (currently 0% with some limited exemptions) would not apply to deregulated organisms, farmers would not need to be subjected to co‐existence restrictions, whose major objective is to prevent adventitious presence and cross‐pollination with regulated GMOs. Again, exclusion of certain organisms from the scope of the legislation would result in a rather broad freedom to use them EU‐wide, whereas an exemption would still allow individual EU member states to introduce local restrictions.

Finally, exclusion or exemption of certain groups of organisms or techniques will also influence international trade with the EU. It will bring EU legislation more in line with the regulatory provisions of some of its major trading partners, who have deregulated such organisms, notably the United States, Canada or Argentina. Without such harmonisation, trade in agricultural products may be hampered by asynchronous or asymmetric authorisation, since gene‐edited plant products not regulated in their countries of origin could still be treated as unauthorised GMOs with zero tolerance policy in the EU. Lack of such harmonisation will also put a strain on the phytosanitary and customs authorities, who will be obligated to prevent the influx of such organisms into the EU while facing objective difficulties with their detection and tracking. Member states, who decide to introduce national limitations on exempted products will have to find reasonable ways to enforce such limitations, which may prove difficult in practice.

## Certainty of law

The disputes on the regulatory status of NBT products originated mostly from the ambiguity of the legislation that led to differences in interpretation. All analysed proposals aim for more clarity and balance in the legislation by excluding or exempting techniques, whose products they consider to be comparable in terms of associated risks with products that are already not GMO‐regulated. The analysed proposals strive to increase the legal certainty and strike a new balance between ensuring a proper level of safety and freedom to conduct research and development. Currently, the weight of the legislation is shifted strongly towards precaution and safety.

Nevertheless, problems may arise from the use of terms with vague or not commonly accepted meanings, such as “closely related species” or “conventional breeding”. Their use, without proper definition, may result in problems not unlike those that are caused by the current wording of the exemptions in the directive. The proposals are drafted by scientists (proposals 1, 2, 4), science students (proposal 5) or experts that likely have a strong scientific background (proposals 3 and 6), who probably find certain terms clearly understandable. However, once put into law, they will be interpreted by lawyers, who might find other ways to interpret them. Would for example rye be considered a closely related species of wheat? Or is randomly induced mutagenesis performed on *in vitro* cell cultures considered conventional? This situation may lead to further disputes. It will also be important to measure the proposals against relevant basic principles of EU law, such as the proportionality principle, the innovation principle and the precautionary principle.

Some of the proposals therefore suggest a pre (the Norway proposal)‐ or post (the Dutch proposal)‐marketing authorisation system limited to confirming the status of a given product. Proposal 6 (Bratlie *et al*, 2019) stands out here, as it provides not only for an expedited authorisation process for intragenic or cisgenic organisms, but also an assessment of “societal benefit, sustainability and ethics” for organisms with heritable changes. The latter solution may lower the certainty of law again by subjecting products to assessment based on non‐scientific criteria. Regardless of these details, a need for a more transparent and less ambiguous authorisation system can be noticed throughout the analysed proposals.

… a need for a more transparent and less ambiguous authorisation system can be noticed throughout the analysed proposals.

## Concluding remarks

It is clear that the analysed proposals pursue similar goals such as easing the administrative burdens connected with the authorisation of some organisms, but they pursue them through different methods. This shows that, at least, the scientific community in the EU shares a common goal even if the proposals are not always compatible with each other in the sense that they either would affect different types of organisms or propose amendments with starkly different consequences. The lack of a common stance from the scientific community or from other stakeholders might therefore increase confusion regarding the preferred way forward.

It is currently unclear, whether the EC will conclude its analysis of the legal status of novel genomic techniques with an inference that a revision of the GMO legislation is in order. *Prima facie*, a revision seems justified given the counter‐intuitive consequences of the current *status quo*—regulating organisms with more controlled genetic changes more strictly than organisms with more randomly introduced genetic changes, such as products of conventional mutagenesis—and the pressure from the scientific community and other stakeholders. Should the EC decide that a revision is necessary, the analysed proposals may indicate the direction in which such a revision might go.

## Conflict of interest

The authors declare that they have no conflict of interest.
